# Understanding the Low Level of Cervical Cancer Screening in Masaka Uganda Using the ASE Model: A Community-Based Survey

**DOI:** 10.1371/journal.pone.0128498

**Published:** 2015-06-01

**Authors:** Cyprian Twinomujuni, Fred Nuwaha, Juliet Ndimwibo Babirye

**Affiliations:** School of Public Health, Makerere University College of Health Sciences, Kampala, Uganda; Kingston University London, UNITED KINGDOM

## Abstract

Cervical cancer is one of the leading causes of cancer deaths among women globally and its impact is mostly felt in developing countries like Uganda where its prevalence is higher and utilization of cancer screening services is low. This study aimed to identify factors associated with intention to screen for cervical cancer among women of reproductive age in Masaka Uganda using the attitude, social influence and self efficacy (ASE) model. A descriptive community based survey was conducted among 416 women. A semi-structured interviewer administered questionnaire was used to collect data. Unadjusted and adjusted prevalence ratios (PR) were computed using a generalized linear model with Poisson family and a log link using STATA 12. Only 7% (29/416) of our study respondents had ever screened for cervical cancer although a higher proportion (63%, 262/416) reported intention to screen for cervical cancer. The intention to screen for cervical cancer was higher among those who said they were at risk of developing cervical cancer (Adjusted prevalence ratio [PR] 2.0, 95% CI 1.60–2.58), those who said they would refer other women for screening (Adjusted PR 1.4, 95% CI 1.06–1.88) and higher among those who were unafraid of being diagnosed with cervical cancer (Adjusted PR 1.6, 95% CI 1.36–1.93). Those who reported discussions on cervical cancer with health care providers (Adjusted PR 1.2, 95% CI 1.05–1.44), those living with a sexual partner (Adjusted PR 1.4, 95% CI 1.11–1.68), and those who were formally employed (Adjusted PR 1.2, 95% CI 1.03–1.35) more frequently reported intention to screen for cervical cancer. In conclusion, health education to increase risk perception, improve women's attitudes towards screening for cervical cancer and address the fears held by the women would increase intention to screen for cervical cancer. Interventions should also target increased discussions with health workers.

## Introduction

Cancer of the cervix is the most common cancer among women in 45 countries, with global reports of more than 500,000 new cases and 270,000 deaths every year [1]. Developing countries report most (85%) of the new cases and 90% of the deaths. In Uganda, for instance, cervical cancer is the most frequent cancer among women with 4000 newly diagnosed cases annually, 80% of which present with advanced disease when cure is impossible [1,2]. In addition, settings with lower prevalence of cervical cancer such as Singapore report that the direct cost of treating invasive cervical cancer could be in excess of 58 million dollars over 25 years [3] which is way above most sub-Saharan African national budgets. Therefore early detection and treatment of precancerous cervical lesions are the most cost-effective interventions for prevention of cervical cancer. However, only 5% of women in developing countries have ever been screened for cervical cancer compared to 84% of their counterparts in developed countries [1,4].

Available evidence indicates that barriers to utilization of screening services could be due to demographic [2,5,6,7] or community characteristics [2,5,6,7] or health system structural barriers [8,9,10]. The Ugandan Ministry of Health strategic plan for cervical cancer prevention and control 2010–2014 aimed at reducing these barriers by targeting dissemination of information about cervical cancer prevention and treatment to 90% of Ugandans, and screening and treatment of 80% of eligible women aged 25–49 years [11]. It is clear that these targets were not met since only the national and regional referral hospitals, selected private not-for-profit and private-for-profit hospitals were equipped to provide cervical cancer screening services [11]. Secondly, these screening centres were managed by the few and highly specialized gynaecologists [9]. Thirdly, earlier Ugandan studies indicate that available cancer screening services were not optimally utilized as the demand for cervical cancer screening was low even at the national referral hospital [8]. This is a cause for concern particularly because the prevalence of HPV virus (the cause of cancer of the cervix) in the East African region is high; estimated at 20% in the general population [12]. It is critical therefore to understand why eligible women do not optimally use available cancer screening services in a setting with one of the highest HPV prevalence in the world. This study used the attitude-social influence-self efficacy (ASE) model to assess factors associated with intention to screen for cervical cancer among women of reproductive age in Masaka, Uganda so as to inform implementation of strategies for prevention and control of cervical cancer.

## Methods

### Study setting

This community based cluster survey was conducted between January and March 2013. The primary objective of the survey was to measure knowledge on cervical cancer among 510 women aged 15–49 years. The objective of this report was to measure intention to screen for cervical cancer and therefore secondary data analysis was conducted on a sub-sample of 416 women aged 25–49 years. Masaka district, where the study was conducted, is located in southern Uganda along the equator, 120km from Kampala the Capital City of Uganda. It is administratively subdivided into 3 counties, 9 sub-counties, 39 parishes and 352 villages. On average each village has 200 households. The district population is approximately 250,000 (projected 2011–12), 52% of whom are females and 11% live in urban areas [13]. Health services in Masaka are provided by two hospitals (both provide cervical cancer services). One of these is the regional referral hospital which serves the population from Masaka and other neighbouring districts. Other general health services are provided by health facilities that provide differing levels of health care services based on the highest qualification of health care workers: three health centre IV, three health centre III and 16 health centre II. In Masaka district cervical cancer data is not routinely analyzed, but a review of Masaka district HMIS 2011–2012 report indicates that 34 cases of cancer of cervix were diagnosed compared to 16 cases of breast cancer in the same year.

### Eligibility and sampling

Respondents were eligible for study inclusion if they were women aged 25–49 years old and were residents of Masaka district. One woman per household was selected for study inclusion. If there was more than one eligible woman; such as a mother and her daughter in the house the older of the two was selected for study inclusion. Women who had confirmed cancer of cervix or total hysterectomy and those who were too sick or were unable to give informed consent were ineligible for study inclusion.

The required sample size for the objective of this report (intention to screen for cervical cancer) was 396 respondents using a modified formula by Bennet et al [14] for cluster surveys with the following assumptions; a two-sided test with a precision of 0.03, 80% power, 30 households per cluster, intraclass correlation of 0.2, design effect of 2.0, proportion of those who have ever screened for cervical cancer of 20% [8] and a non-response rate of 10%.

A multistage sampling technique was employed for the selection of study participants. In the first stage one of three counties in Masaka district was randomly selected using computer generated random numbers. Then four of the 9 parishes in Masaka district were randomly selected using random numbers. The number of respondents at each parish was determined using sampling proportionate to the population size of women estimated for each parish using the population projection for 2010 from the Uganda Bureau of Statistics [13]. A village was considered a cluster and these were randomly selected from each parish using computer generated random numbers.

At the last stage selection of households was conducted in the following manner; a central point in a selected village was chosen and starting from the household in the western direction, research assistants moved from house to house interviewing eligible respondents until the required sample for that village was obtained (30 respondents). In case a respondent declined to participate or was not home at the time the house was approached the next household was considered for study inclusion.

### ASE model

**Fig 1:**
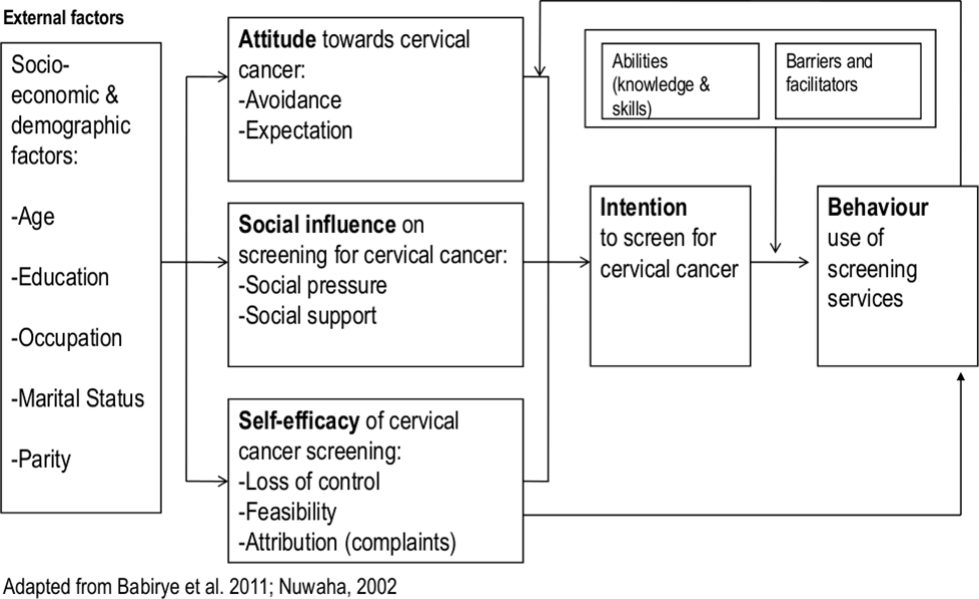
Attitude-Social Influence-Self-efficacy Model.

Data on the intention to screen for cervical cancer and the factors associated with this intention was collected based on the attitude-social influence-self efficacy model (ASE, shown in Fig 1). This model was originally developed by de Vries et al for smoking cessation [15]. The model was selected for use in this study because it considers both social influence and self efficacy as predictors of behaviour. The health belief model as well as the trans-theoretical model consider self-efficacy but do not consider social influence as a predictor of behaviour. Besides the trans-theoretical model is focused on promoting change in behaviour [16] whereas as the ASE model is better suited to explaining current behaviour.

In the ASE model presented in this paper behaviour related to using cervical cancer screening services is directly determined by the behaviour intention. This intention is in turn influenced by three main psychosocial factors; the attitude, social influence, and self efficacy. A person's attitude refers to the extent to which a person has a favourable or unfavourable evaluation of the behaviour. A person's attitude towards cervical cancer screening may be influenced by personal beliefs such as misconceptions associated with cancer of the cervix, and by the fear associated with the screening procedure. This fear is a barrier to optimal utilization of screening services [17,18,19].

Social influence is a process where people directly or indirectly influence thoughts, feelings and action of others [19]. It results from social norms related to cervical cancer screening and the support from important others like the spouse or in-laws. Self efficacy refers to a person's perceived ability to cope with barriers that may hinder adherence to recommended cancer screening schedules. A low perceived benefit of cervical cancer screening would reduce the perceived ability to cope with the barriers to screening. Self efficacy not only influences behaviour intention but also directly influences behaviour. Barriers and abilities could influence use of cervical cancer screening services. Previous behaviour or trying to perform the behaviour has a feedback mechanism that in turn influences the attitude, social influence and self efficacy. In public health activities, the demographic characteristics are not changeable thus the focus by the ASE model on attitudinal, social influence and self- efficacy variables.

### Measurements

The socio-demographic characteristics measured in this study are shown in Table 1. Intention to screen was measured by three questions; do you wish to undergo cervical cancer screening? when do you intend to go for screening? Give reasons for your response? (see variables in Table 2). The factors associated with the intention to screen for cervical cancer were assessed and categorized into attitudinal, social influence, and self efficacy (ASE) factors based on the ASE model described above. Questions assessing the attitude of respondents towards cervical cancer screening included those on the fear of cervical cancer screening procedure, side effects of the procedure, vaginal examinations, or a diagnosis of cervical cancer. Respondents were also asked if they would refer other women for cervical cancer screening services, Table 3. Social influence was assessed by asking respondents if they had discussed cervical cancer and its screening with important others including spouses, close relatives or peers; the response from important others and the outcome of the discussions; and the decision making process for cervical cancer screening were also examined, Table 4. Questions on self efficacy focused on the perceived ability to overcome health system barriers such as availability of cervical cancer screening services, distance to the facility where services were provided, privacy issues at the facility and costs incurred in seeking services, Table 5.

**Table 1:** Socio-demographic characteristics of the respondents.

Variable	Frequency	Percent
**Age of Respondents**		
25–39 years	285	31.5
40–49 years	131	68.5
**Years completed in formal education**		
0–7 years	294	70.7
8–13+ years	122	29.3
**Living with a partner**		
Yes	286	68.8
No	130	31.2
**Type of relationship for those living with partners(n = 286)**		
Monogamous	133	46.5
Polygamous	153	53.5
**Formally employed**		
Yes	174	41.8
No	242	58.2
**Number of children** ^**1**^		
0–1 children	50	12.0
2–3 children	146	35.2
4+ children	219	52.8

^1^One person had missing data for this variable. In addition, 25 respondents had never had children

**Table 2:** Utilization of cervical cancer screening services.

Variable	Frequency	Percent
**Have you ever screened for cervical cancer?** (n = 416)		
Yes	29	7.0
No	387	93.0
**Number of screening tests ever done** (n = 29)		
Once	23	79.3
Twice	4	13.8
Three times	2	6.9
**When was the last screening test done?** (n = 29)		
Less than one year	4	13.8
One year to Three years ago	15	51.7
Over three years ago	10	34.5
**Do you intend to screen for cervical cancer in the future?** (n = 416)		
Yes	262	63.0
No	154	37.0
**When do you intend to screen?** (n = 254)^1^		
Within one month’s time	23	9.1
In two months time	68	26.8
After one year	138	54.3
After two years	25	9.8

^1^Data was missing for 8 respondents

**Table 3:** Demographic characteristics and intention to screen for cervical cancer-Univariable analysis.

	Intention to screen			
	Yes	No		
Variable	n = 262 (%)	n = 154 (%)	Unadjusted PR(95%CI)	p- value
**Age of respondent**				
25–39 years	193 (67.7)	92 (32.3)	1.3 (1.07–1.54)	0.004
40–49 years	69 (52.7)	62 (47.3)	1	
**Year completed in formal education**				
0–7 years	185 (62.9)	109 (37.1)	1.0 (0.84–1.17)	1.000
8–13+ years	77 (63.1)	45 (36.9)	1	
**Living with a sexual partner**				
Yes	210 (73.4)	76 (26.6)	1.8 (1.47–2.29)	0.001
No	52 (40.0)	78 (60.0)	1	
**Type of relationship for those living with sexual partner (n = 286)**				
Monogamous	100 (75.2)	33 (24.8)	1.0 (0.91–1.20)	0.592
Polygamous	110 (71.9)	43 (28.1)	1	
**Formally employed**				
Yes	131 (75.3)	43 (24.7)	1.4 (1.20–1.61)	0.001
No	131 (54.1)	111 (45.9)	1	
**Number of children (n = 415)**				
0–1 children	23 (46.0)	27 (54.0)	1	
2–3 children	106 (72.6)	40 (27.4)	1.6 (1.15–2.17)	0.001
4+ children	132 (60.3)	87 (39.7)	1.3 (0.95–1.80)	0.090

**Table 4:** Attitude and intention to screen for cervical cancer-univariable analysis.

	Intention to screen			
	Yes	No	Unadjusted	
Variable	n = 262 (%)	n = 154 (%)	PR (95%CI)	p- value
**Do you think you are at risk of developing cervical cancer?**				
Yes	216 (76.1)	68 (23.9)	2.2 (1.71–2.78)	0.001
No	46 (34.8)	86 (65.2)	1	
**The respondent fears the screening procedure**				
Yes	170 (67.2)	83 (32.8)	1.2 (1.01–1.40)	0.029
No	92 (56.4)	71 (43.6)	1	
**The respondent is afraid of discomfort during and after the procedure**				
Yes	54 (68.4)	25 (31.6)	1.1 (0.93–1.32)	0.302
No	208 (61.7)	129 (38.3)	1	
**The respondent is afraid of pain during and after the procedure**				
Yes	141 (66.2)	72 (33.8)	1.1 (0.96–1.29)	0.187
No	121 (59.6)	82 (40.4)	1	
**The respondent is afraid of bleeding following the procedure**				
Yes	93 (64.1)	52 (35.9)	1.0 (0.88–1.20)	0.750
No	169 (59.6)	102 (37.6)	1	
**The respondent is afraid of vaginal examinations**				
Yes	38 (57.6)	28 (42.4)	0.9 (0.72–1.12)	0.333
No	224 (64.0)	126 (36.0)	1	
**Do you have any fear of being diagnosed with cervical cancer?**				
Yes	205 (61.2)	130 (38.8)	1.1 (0.97–1.34)	0.097
No	57 (70.4)	24 (29.6)	1	
**Do you prefer to receive services from male or female service providers?**				
Female	97 (62.6)	58 (37.4)	1	
Male	33 (82.5)	7 (17.5)	1.3 (1.09–1.59)	0.023
None	132 (59.7)	89 (40.3)	1.0 (0.81–1.12)	0.593
**Would you refer other women for screening services?**				
Yes	240 (65.9)	124 (34.1)	1.6 (1.13–2.16)	0.002
No	22 (6.7)	30 (93.2)	1	
**Have you ever screened for cervical cancer?**				
Yes	21 (72.4)	8 (27.6)	1.2 (0.92–1.47)	0.323
No	241 (62.3)	146 (37.7)	1	

**Table 5:** Social influence and intention to screen for cervical cancer-univariable analysis.

	Intention to screen			
	Yes	No		
Variable	n = 262 (%)	n = 154 (%)	Unadjusted PR (95%CI)	p-value
**Have you ever discussed cervical cancer with your spouse?**				
Yes	41 (80.4)	10 (19.4)	1.3 (1.13–1.56)	0.005
No	221 (60.5)	144 (39.5)	1	
**Have you ever discussed cervical cancer with a Village Health Team (VHTs) member?**				
Yes	12 (75.0)	4 (25.0)	1.2 (0.90–1.61)	0.431
No	250 (62.5)	150 (37.5)	1	
**Have you ever discussed cervical cancer with close relatives?**				
Yes	21 (67.7)	10 (32.3)	1.1 (0.84–1.40)	0.700
No	241 (62.6)	144 (37.4)	1	
**Have you ever discussed cervical cancer with peers?**				
Yes	32 (58.2)	23 (41.8)	0.9 (0.72–1.16)	0.455
No	230 (63.7)	131 (36.3)	1	
**Would seek permission before seeking screening services?**				
Yes	213 (65.3)	113 (34.7)	1.2 (0.98–1.47)	0.065
No	49 (54.4)	41 (45.6)	1	

A pre-tested semi-structured interviewer administered questionnaire was used to collect the data. The questionnaire was developed using questions from previously published surveys [2,4,5,9,10,20,21,22,23], from key messages on the WHO website [24] and from validated tools. These tools report high internal reliability for total cervical cancer knowledge with Cronbach's α of >0.8 in the United Kingdom [25,26]. Validation is yet to be done in African settings. Consequently 62 questions were created for our questionnaire and these covered a range of topics including cervical cancer screening, awareness (including warning signs and risk factors), reproductive history of the respondents, and socio-demographic characteristics. The internal reliability of our scale was estimated using Cronbach's α for 7 questions related to fear of cancer screening procedure, vaginal examination, or cancer diagnosis, seeking permission from others before going for the screening test, discussions on cervical cancer with the spouse, being encouraged to screen by a health worker and having previously screened for cervical cancer. This gave a Cronbach's α of 0.75.

Each interview lasted about 60 minutes and each of the three female data collectors (one midwife, one nurse, and one health educator) conducted about 5 interviews per day. The questionnaire was translated into the local language (Luganda) and back translated into English for consistency in meaning. Interviews were conducted in the local language. The questionnaires were reviewed daily for completeness and corrections made where necessary.

### Data management and analysis

Data was edited, cleaned, entered and analyzed using STATA version 12 software. To measure the association between the primary outcome (intention or no intention to screen for cervical cancer) and the ASE factors, we used Prevalence Ratios (PR) rather than Odds Ratios because the primary outcome was highly prevalent (>10%). Odds Ratios tend to overestimate the strength of association in such cases [27]. The prevalence ratios were computed using a generalized linear model with Poisson family and a log link with robust standard errors using a backward elimination method. We present unadjusted and adjusted PRs plus their 95% confidence interval (95% CI) and p values at α = 0.05Simple models were done for each of the variables before the multivariable model was developed. Multivariable modeling was done to optimally predict who will and who won’t intend to screen. At this stage variables that had a p value <0.20 at univariable analysis or if their inclusion resulted in a change of 10% or more of the PR and those that were biologically plausible were included in the model to identify independent factors associated with intention to screen for cervical cancer while controlling for confounding and interaction by other factors.

### Ethics

Ethics approval was obtained from Makerere University School of Public Health Higher Degrees Research and Ethics Committee and independently from the Uganda National Council for Science and Technology. Permission to conduct the study was also obtained from the District health office in Masaka. Study participants provided informed written consent on a participant’s consent form approved by the ethics bodies.

## Results

A total of 510 women were approached for study inclusion and all consented to study participation for the primary objective of measuring cervical cancer awareness. The final sample considered for secondary data analysis for this report was 416/510 (81.6%) and this included women aged 25–49 years with a mean age of 35.7 years (SD = 7.3) and a median of 35 years. Other respondent characteristics are shown in Table 1 below.

### Use of cervical cancer services

Most (85.8%, 357/416) respondents had heard about cervical cancer. Only 7.0% (29/416) had ever screened for cervical cancer however. Most of these had screened once (79.3%, 23/29) and 34.5% (10/29) had screened more than three years prior to the study. Intention to go for cervical cancer screening was reported among 63.0% (262/416) of the respondents and only 5.5%% (23/416) intended to screen within one month’s period (Table 2). At univariable analysis (Table 3), age 25–39 years, living with a partner, having 2–3 children and formal employment were statistically associated with intention to screen for cervical cancer (p<0.05).

### Attitudinal factors

More than half (68.3%, 284/416) of the respondents said they were at risk of cervical cancer. Most (76.1%, 216/284) women who considered themselves at risk of cervical cancer intended to screen compared to 34.8% (46/132) who did not consider themselves at risk (unadjusted prevalence ratio (PR) 2.2, 95% CI 1.71–2.78). Among those who did not intend to screen for cervical cancer, one third (29.9%, 46/154) reasoned that they did not have symptoms for cervical cancer and 18.2% (28/154) reported that they were not at risk of cervical cancer. Respondents reported fear of pain (51.2%, 213/416), bleeding (34.9%, 145/416) and discomfort (19.0%, 79/416) during and after the procedure; however, these were not statistically associated with intention to screen at univariable analysis. More than half (60.8%, 253/416) of the respondents had fears towards the procedure for cervical cancer screening. More women (65.9%, 240/364) who reported that they would refer other women for screening services compared to 6.7% (22/326) of those who would not refer others more often reported intention to screen for cervical cancer (unadjusted PR 1.6, 95%CI 1.13–2.16). Also most (82.5%, 33/40) women who preferred male service providers compared to 62.6% (97/155) of those who preferred female service providers (unadjusted PR 1.3, 95%CI 1.09–1.59) reported intention to screen for cervical cancer (Table 4). However, only 9.6% (40/416) of the respondents preferred male service providers.

### Social influence

About one third (28.1%, 117/416) of the respondents reported that they would consult others before deciding to screen for cervical cancer. A very small proportion of women in this study had ever discussed cervical cancer with spouses (12.3%, 51/416), close relatives (7.5%, 31/416), peers (13.2%, 55/416), and with VHTs (3.8%, 16/416). More (80.4%, 41/51) women who reported discussions with the spouse intended to go for cervical cancer screening compared to 60.5% (250/400) of women who reported no discussions (unadjusted PR 1.3, 95%CI 1.13–1.56), Table 5.

### Self efficacy factors

The perceived ability to cope with health system barriers were considered for this study. More than half (67.1%, 279/416) of the respondents knew where cervical cancer screening services were offered. The distance to the nearest cervical cancer screening centre ranged from 2km to 45km and only half (46.7%, 129/276) lived within 20km from the nearest screening health facility. This meant that transport costs to the facility were relatively high ranging from Uganda shillings 3000 (equivalent to US$ 1.2) to Uganda shillings 30,000 (equivalent to US$ 12) with an average expenditure of Uganda shillings 13,000 (equivalent to US$ 5.2). Although screening services are free in Uganda, costs particularly for cervical cancer screening services were reported by 4.6% (19/416) of the respondents. The cost for services ranged from Uganda shillings 3000 (equivalent to US$ 1.2) to 100,000 (equivalent to US$ 40) with an average cost of Uganda shillings 43,000 (equivalent to US$ 17.2). The total costs incurred for services were reportedly prohibitive for service utilization among 89.7% (174/194) of the respondents although this was not statistically significant (unadjusted PR 0.9, 95% CI 0.67–1.20). Concerns for privacy at the screening centre were a barrier for intention to screen for cervical cancer for more than half (48.6%, 129/333) of the respondents. Most (70.4%, 162/230) women who reported discussions with health workers intended to go for screening compared to 53.8% (100/186) who reported no discussions (unadjusted PR 1.3, 95%CI 1.12–1.53), Table 6. Although the numbers of those that had previously used screening services were small (29/416), use of services was associated with respondent' reports that cervical cancer screening services were offered by health workers during visits to the health facility for other reasons (unadjusted PR 3.0, 95%CI 1.24–7.24) and reports of discussions on cervical cancer with health workers (unadjusted PR 10.9, 95%CI 2.63–45.31); but not with the distance to the nearest health facility that offers screening services (unadjusted PR 1.7, 95%CI 0.80–3.44), transport costs (unadjusted PR 0.9, 95%CI 0.41–2.00) nor privacy concerns (unadjusted PR 1.3, 95%CI 0.63–2.59).

**Table 6:** Self-efficacy and intention to screen for cervical cancer-univariable analysis.

	Intention to screen			
	Yes	No	Unadjusted	
Variable	n = 262 (%)	n = 154 (%)	PR (95% CI)	p-value
**Cervical cancer screening services are offered by health workers during other services**				
Yes	21 (77.8)	6 (22.2)	1.3 (1.00–1.56)	0.148
No	241 (62.0)	148 (38.0)	1	
**Have you ever discussed cervical cancer with health workers?**				
Yes	162 (70.4)	68 (29.6)	1.3 (1.12–1.53)	0.001
No	100 (53.8)	86 (46.2)	1	
**What is the distance from your home to the nearest cancer screening centre? (n = 276)** ^**1**^				
Up to 20km	85 (65.9)	44 (34.1)	1	
Above 20km	114 (77.6)	33 (22.4)	1.2 (1.01–1.37)	0.033
**Would privacy be compromised during cervical cancer screening?**				
Yes	144 (67.9)	68 (32.1)	1.2 (1.01–1.36)	0.042
No	118 (57.8)	86 (42.2)	1	
**Transport costs to the cancer screening centre (n = 248)** ^**2**^				
≤ shs. 10,000	58 (68.2)	27 (31.8)	1	0.662
> 10,000	116 (71.2)	47 (28.8)	1.0 (0.88–1.24)	
**Respondents reported that the costs for transport and services hinder service usage (n = 194)** ^**3**^				
Yes	111 (63.8)	63 (36.2)	0.9 (0.67–1.204)	0.762
No	14 (70.0)	6 (30.0)	1	

^1^Unequal missing data 24.0% vs. 50.0% column percentages considered

^2^Unequal missing data 33.6% vs. 51.9% column percentages considered

^3^Unequal missing data-53.3% vs. 55.2% column percentages considered

### Independent factors

At multivariable analysis, there were three attitudinal factors independently associated with intention to screen for cervical cancer. For instance, the prevalence of intention to screen for cervical cancer was two times higher among respondents who said they were at risk of developing cervical cancer compared to those who had a low risk perception (Adjusted PR 2.0, 95% CI 1.60–2.58); the prevalence was also 40% higher among those who said they would refer other women for screening (Adjusted PR 1.4, 95% CI 1.06–1.88) and 60% higher among those who said they were unafraid of being diagnosed with cervical cancer (Adjusted PR 1.6, 95% CI 1.36–1.93). Even though only half (55.3%, 230/416) of our respondents had had discussions on cervical cancer with health workers, those who reported discussions on cervical cancer with health care providers (Adjusted PR 1.2, 95% CI 1.05–1.44) more often reported intention to screen for cervical cancer. There were two demographic variables associated with intention to screen for cancer; marital status and occupation of the respondents. The prevalence of intention to screen was 40% higher among those who reported that they were living with a sexual partner compared to those that were not (Adjusted PR 1.4, 95% CI 1.11–1.68), and 20% higher among the formally employed compared to the unemployed (Adjusted PR 1.2, 95% CI 1.03–1.35), Table 7.

**Table 7:** Independent predictors of intention to screen for cervical cancer.

Variables in the multivariable analysis	Unadjusted PR (95% CI)	AdjustedPR (95% CI)
**Do you think you are at risk of developing cervical cancer?**		
Yes	2.2 (1.71–2.78)	2.0 (1.60–2.58)
No	1	1
**Do you have any fear of being diagnosed with cervical cancer?**		
Yes	1.1 (0.97–1.34)	1.6 (1.36–1.93)
No	1	1
**Do you have any fear towards the screening procedure?**		
Yes	1.2 (1.01–1.40)	1.0 (0.86–1.27)
No	1	1
**Do you prefer to receive services from male or female service providers?**		
Female	1	1
Male	1.3 (1.09–1.59)	1.1 (0.95–1.38)
None	1.0 (0.81–1.12)	1.0 (0.88–1.19)
**Would you refer other women for screening services?**		
Yes	1.6 (1.13–2.16)	1.4 (1.06–1.88)
No	1	1
**Have you ever discussed cervical cancer with health workers?**		
Yes	1.3 (1.12–1.53)	1.2 (1.05–1.44)
No	1	1
**Cervical cancer screening services are offered by health workers during other services**		
Yes	1.3 (1.00–1.56)	1.1 (0.84–1.31)
No	1	
**Would privacy be compromised during cervical cancer screening?**		
Yes	1.2 (1.01–1.36)	1.0 (0.82–1.10)
No	1	1
**Living with a sexual partner**		
Yes	1.8 (1.47–2.29)	1.4 (1.11–1.68)
No	1	
**Formally employed**		
Yes	1.4 (1.20–1.61)	1.2 (1.03–1.35)
No	1	1
**Age of respondent**		
25–39 years	1.3 (1.07–1.54)	1.1 (0.91–1.29
40–49 years	1	1
**Number of children**		
0–1 children	1	1
2–3 children	1.6 (1.15–2.17)	1.3 (0.94–1.69)
4+ children	1.3 (0.95–1.80)	1.2 (0.92–1.64)
**Have you ever discussed cervical cancer with your spouse?**		
Yes	1.3 (1.13–1.56)	1.0 (0.85–1.15)
No	1	
**Would seek permission before seeking screening services?**		
Yes	1.2 (0.98–1.47)	1.1 (0.88–1.30)
No	1	1

## Discussion

This study assessed attitudinal, social influence and self-efficacy factors associated with intention to screen for cervical cancer. Only 7% of the study respondents had ever screened for cervical cancer and 63% intended to go for cervical cancer screening. The prevalence for intention to screen for cervical cancer was higher among those who reported risk perception for cervical cancer, those who were unafraid of being diagnosed with cervical cancer and those that would refer other women for screening. The prevalence of intention to screen for cervical cancer was also significantly higher among respondents who reported discussions with health workers, those living with sexual partners and those who are formally employed.

One of the major findings of our study was the very low proportion of women that had ever screened for cervical cancer. Similarly low proportions (7%) were reported among rural Indian women [21] and surprisingly among health workers in Nigeria (6%) [22], India (12%) [28], 2013) and Uganda (19%) [8]. The low proportion of service use despite professional background may be attributed to a low cervical cancer risk perception among eligible service users as shown in our study and also similar to findings elsewhere [6,8,28]. Another reason that may explain the low level of intention to screen for cervical cancer is the fear of cervical cancer diagnosis. Studies in other developing countries report similar attitudinal barriers related to the screening procedure or vaginal examinations [6,28] which may explain why women usually present with late stage disease when the cure for cervical cancer is practically impossible [2].

In addition to the low level of cervical cancer screening service usage, those that used the service reported irregular service use. According to the strategic plan for cervical cancer prevention and control in Uganda 2010–2014, it is recommended that sexually active women should be screened for cervical cancer at least once every two years and more often for HIV positive women [11]. Our study findings show that guidelines were not adhered to since one third of the respondents had last screened for cervical cancer more than three years prior to the study. Similarly irregular service use were reported in the Tanzanian [6] and Indian [28] studies mentioned earlier. Non-adherence to guidelines is not limited to use of cervical cancer screening services however but has been reported in other health programs in Uganda [29].

Social influence from important others such as the spouse played a significant positive role in intention to screen for cervical cancer in our study. Emphasizing the need to increase male involvement in cancer screening services [6,30,31] because the lack of male involvement is reportedly prohibitive for successful health programs [9,30,31]. In addition, health systems in place have to be adjusted to accommodate men since the current systems at many health facilities are oriented toward women to the extent that they have become institutional barriers to greater male involvement [17,31]. The influence of men on health seeking by women cannot be overemphasized in a developing setting such as Uganda since it is often related to the influence of hierarchy and power between men and women which underlies several aspects of decision making for health [31].

Only 10% of our study respondents preferred male service providers although, those who preferred male service providers had a higher prevalence for intention to screen for cervical cancer compared to those who preferred female service providers at univariable analysis. It is unclear why our findings contrast reports from other places [23] which have found that the preference for female health workers is due to privacy concerns because of the posture (lithotomy position) women have to take during pap smears in hospitals [9,23]. Privacy concerns were a barrier to intention to screen for about half of our study respondents although this was significant at univariable analysis only. In Uganda, pap smears are mostly provided by the highly specialized gynaecologists at referral health facilities and most of these are males [8,9]. It is critical that we design programmes that are accessible and acceptable to the general population in order to improve utilization of cancer screening services. To further emphasize the need to remove all health system structural barriers, our study found that respondents who reported discussions on cervical cancer with health workers more frequently reported intention to screen for cervical cancer. However, only about half of the respondents reported discussions with health workers. Therefore training of health workers should aim at increasing suspicion of cancer of the cervix among eligible women [8,9,10].

### Methodological considerations

Our study was based on the ASE model to explore factors associated with intention to screen for cervical cancer. The ASE model has previously been used for studies on uptake of voluntary counselling and testing for HIV [18] and partner referral to screen for sexually transmitted infections [32]. Inconsistent with the ASE model utilized in this study, some of the demographic characteristics of the respondents were associated with intention to screen for cervical cancer. Although, marital status may be indicative of discussions and support of men in the spouse’s decision making for health. Furthermore, previous cancer screening had no association with intention to screen for cervical cancer which may be related to a very low proportion of previous screening behaviour in this setting. The ASE model has been useful in identifying possible factors associated with intention to screen for cervical cancer in this study. However, like all other cross sectional studies, the direction of causality could not be established. One of the strengths of this study was that data collection was conducted at the household level which included participants who were unable to overcome barriers to cervical cancer screening. In addition, the sampling procedure used in this study could have introduced some bias as the selected sample was from one county in Masaka district. However, our findings may have implications for cervical cancer programmes in similar settings since they are similar to those from other parts of Uganda and other developing countries as already described in the discussion above. Lastly, although HIV status of the respondents could influence intention to screen for cervical cancer, this was not measured in our study.

## Conclusions

Although more than half (63%) of our study respondents reported intention to screen for cervical cancer, only 6.5% were ever offered that opportunity by health care workers, which may partly explain why the uptake of screening services was only 7% in this rural setting. The level of intention to screen may be related to a low risk perception towards cervical cancer and fear of cervical cancer diagnosis in our study population similar to that reported in other African populations. Health education may increase the population risk perception and address the fears held by the women. Social influence on intention to screen for cervical cancer was observed among those who lived with sexual partners. The implication is that health workers need to target male partners with information on cervical cancer and its prevention in order to increase the intention to screen for cervical cancer. Lastly, discussions with health workers specifically targeting the unemployed would increase intention to screen for cervical cancer.
